# Beyond Controversy? The Promotion and Early Critical Reception of *Sociobiology: The New Synthesis*, Fifty Years Later

**DOI:** 10.1007/s10739-025-09848-1

**Published:** 2026-01-08

**Authors:** Cora Stuhrmann

**Affiliations:** Ludwig-Maximilians-Universität München, München, Germany

This year marks the fiftieth anniversary of the publication of E.O. Wilson’s *Sociobiology: The New Synthesis* (Wilson 1975). It is almost impossible to separate the book from the controversy surrounding it. Over the last five decades the initial controversy invited manifold interpretations of what was truly at stake: whether it was nature versus nurture, free will versus determinism, liberal politics versus radical leftism, or reactionary reductionism versus liberatory dialectics. In the efforts to get to the core of the matter, dichotomies abounded. Throughout these debates the term “sociobiology” took on multiple meanings. It can now be used to convey a position in the nature-nurture debate that emphasizes nature, or in the two-culture debate that emphasizes science, or in the gene-culture debate that emphasizes genes; more generally it is used to refer to the concept of human nature based on biology. It is not often used as a self-identification but rather to identify the opposing stance. This is done because the term also conveys the limitations of this position, standing *pars pro toto* for the dangers of biological determinism, scientific reductionism, and disciplinary imperialism.

But the term can also refer to Wilson’s unilateral attempt to establish a discipline of the same name, which was “defined as the systematic study of the biological basis of all social behavior” (Wilson 1975, p. 4).^1^ This meaning is further complicated by its extension to other efforts with diverse intellectual starting points in the biological and social sciences. Several journals established around *Sociobiology’s* publication show the diverging disciplinary ranges the term can refer to. The narrowest is exemplified by the journal *Sociobiology*, which was established in 1975 and covers social insects; *Behavioral Ecology and Sociobiology* was established in 1976 and has a broader focus on animal behavior; and *Sociobiology and Ethology* was established in 1979 and dedicated to “studies where the primary emphasis is man” (Blurton Jones and McGuire 1979, p. 1).^2^ Additionally, there are other branches of the field now called evolutionary social or evolutionary behavioral science that might be described as sociobiology. However, researchers from the field rarely invoke this term for their work and would describe themselves as evolutionary psychologists, human behavioral ecologists or human ethologists, or working on cultural evolution or gene-culture co-evolution. When used as a disciplinary term it is also more often employed as an external reference rather than a self-identification.

Sociobiology as a term today contains many shifting meanings and its disciplinary reference is heavily dependent on the concrete context. Rather than referring to one clearly defined discipline as Wilson originally intended, it now evokes ambiguity. The multitude of meanings invites us to reflect: What do we mean when we use the term sociobiology? And how and when did the term acquire so many particular meanings? Finally, who precisely are we referring to, when we speak of sociobiologists? While the term sociobiology was in usage before it was associated with the publication of Wilson’s book in 1975, its meanings were largely shaped by the reception and controversies surrounding it. These generated long-term multi-disciplinary debates about determinism, reductionism and the intractable problem of human nature. Concurrently, disciplinary developments on the border between biology and social science occurred in *Sociobiology’s* wake.

The entire history of the sociobiology controversies or debates cannot be recounted here. Yet the intricate history of sociobiology’s ambiguous disciplinary meanings can at least be anchored in the single case where we can unambiguously call something *Sociobiology*: the eponymous monograph. The disciplinary ambiguity of sociobiology we can observe today is rooted in the book itself. Wilson wanted it to make more than a regular scholarly contribution; he wanted it to be the foundational text for a new discipline, a unified science of social behavior. The disciplinary ambition Wilson had attached to *Sociobiology* provides an alternative perspective for examining its conceptualization, promotion, and reception, not as a controversy or debate, but focused on its goal of laying the foundation for a discipline.

*Sociobiology* was initially conceived as a textbook on animal social behavior, but its ambition was soon inflated to become more attractive to lay readers, declaring a new discipline with relevance for human behavior. This inflation of ambition was accentuated by a strategic press campaign that presented a public image of a theoretically solidified discipline of human and animal behavior. The scientific reception of the book, however, was far from uncritically positive, and deflated these ambitions: Sociobiology did not in fact represent a unified discipline with a distinct theoretical foundation and was not mature enough “to reformulate the foundations of the social sciences” (Wilson 1975, p. 4). When contrasting the promotion and reception of *Sociobiology* we can discern how its disciplinary ambition succumbed to disciplinary ambiguity.

## “Not Just a Conventional Textbook” – *Sociobiology’s* Composite Nature

**Fig. 1:**
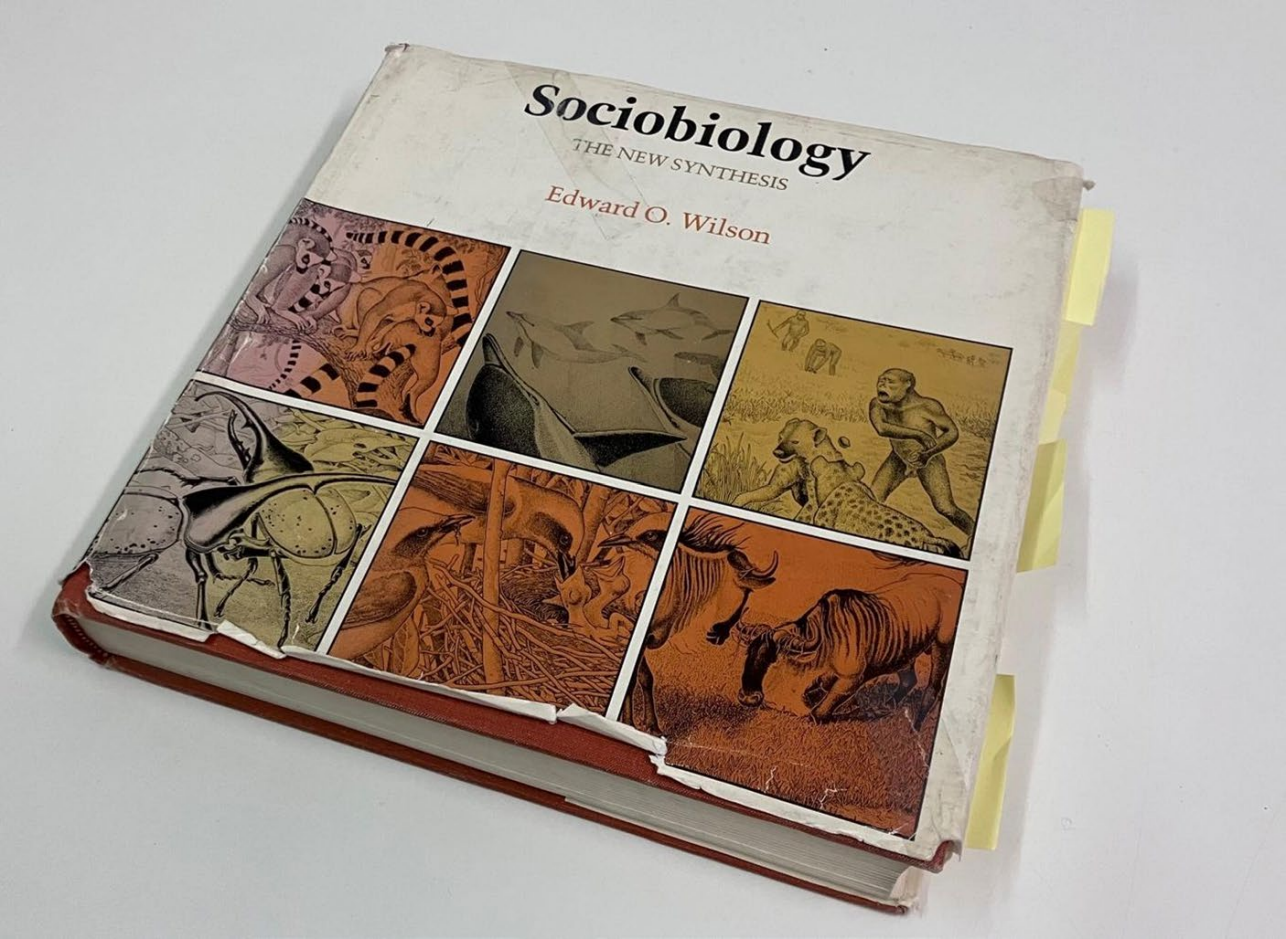
Photograph of the author’s copy of the second printing of *Sociobiology: The New Synthesis* (Cambridge, Mass.: The Belknap Press of Harvard University Press 1975). Copyright © 1975 by the President and Fellows of Harvard College. Used by permission. All rights reserved.

*Sociobiology* seems to fall into the category of what Erika Milam described as a “colloquial science book” that became popular in the 1960s and was written in a “style intended to engage readers only passingly familiar with his (or her) subject” (Milam 2019, p. 4). Previous authors such as Konrad Lorenz, Robert Ardrey, and Desmond Morris had employed this style to write popular works on questions of human nature (Milam 2019; Weidman 2021). Thematically Wilson’s exploration of the evolutionary basis of social behavior overlapped with their accounts of aggression, territoriality and sexual behavior in human pre-history. Like them, Wilson also intended *Sociobiology* for different publics: laymen, scientists with expertise in other fields, especially the social sciences, and his biological colleagues. But where these earlier works appealed to a general interest reader in format and style, *Sociobiology* revealed its composite nature in its format. Published as an almost perfectly square coffee table book rather than a standard trade hardcover size, it did not encourage casual reading, but instead was associated with ownership, display, or serious study. Many reviewers noted the unusual format (25 cm×26 cm) and some even the weight (2.5 kg), which were “dimensions worthy of the Gutenberg Bible,” and made for “strenuous reading” (van den Berghe 1976, p. 733). The book’s immense size – its heft remarked upon in pounds rather than pages – meant it was not particularly appealing for “light bedtime reading,” or fit for re-release as a paperback, but instead more suitable for chapter by chapter reading assignment in a college classroom (Fig. 1).^3^

Additionally, for most of the book Wilson’s writing was not colloquial. Throughout most of its twenty-six chapters, he retained a technical style with extensive references and uncompromising scientific jargon. While Wilson added a glossary for a broad audience, he also conceded that “rapid comprehension” relied on a college-level course in biology and the “most technical chapters” required training in calculus and probability theory (Wilson 1975, p. 577).^4^ Sarah Landry’s famous detailed illustrations only appeared in the last third of the book while the first two sections “Social Evolution” and “Social Mechanism” were dominated by numerous models and formulae, long tables and technical diagrams. Only the first chapter titled “Morality of the Gene,” and to a lesser extent the last chapter titled “Man: From Sociology to Sociobiology,” employed the readable scientific prose that Wilson’s later popularizations became known for. While these chapters were most clearly aimed at colloquial readers, the book did not develop one singular clear-cut message over its many chapters, unlike the works of Lorenz or Ardey before, or those of Richard Dawkins afterwards. These books also summarized their main claim in an evocative or provocative title such as *The Selfish Gene* or *The Territorial Imperative*, whereas *Sociobiology: The New Synthesis* was a rather cumbersome title with its subtitle an allusion to the “Modern Synthesis” that most colloquial readers would be unfamiliar with. In short, in format and style *Sociobiology* was more textbook than popular bestseller.

Indeed, Wilson had initially pitched the book to Harvard University Press (HUP) as a college textbook and scholarly compendium entitled *The Biology of Societies* with the potential for a “shorter, more popular version” to be released later.^5^ By early 1974, a new strategy replaced this clear separation of content and audience: the book was to be marketed to both, the general reader and scientific audiences. Therefore, Harvard University Press suggested a new title in a letter. “I’ll buy Sociobiology,” Wilson replied, but allowed for a subtitle only “if it is not redundant or mere embellishment. One that appeals to me is *Sociobiology: The New Synthesis*. This says that the book is not just a conventional textbook.”^6^ Rather than provide a neutral description of contents, this new title also emphasized the disciplinary ambitions Wilson had harbored for some time.

In previous publications he had already promoted the idea of a “unified science of sociobiology.” There, he presented sociobiology as the expected outcome from merging population and behavioral biology with the proclaimed disciplinary goal to “predict features of social organization” from population parameters based on evolutionary history and ecology.^7^ The new title, *Sociobiology: The New Synthesis* signified Wilson’s proclamation of a new discipline. Rather than to merely summarize existing empirical findings or elucidate established theories to students and colleagues inside the biological field, Wilson wanted to persuade them of his disciplinary vision, one that was not only built on biology, but also meant to incorporate the social sciences. The composite nature of the book united three already vast ambitions: to have the broad appeal of a scientific bestseller, to provide the extensive content of a specialist compendium, and to function as a foundation for disciplinary developments that stress the relevance of biology for the study of human social behavior.

**Fig. 2:**
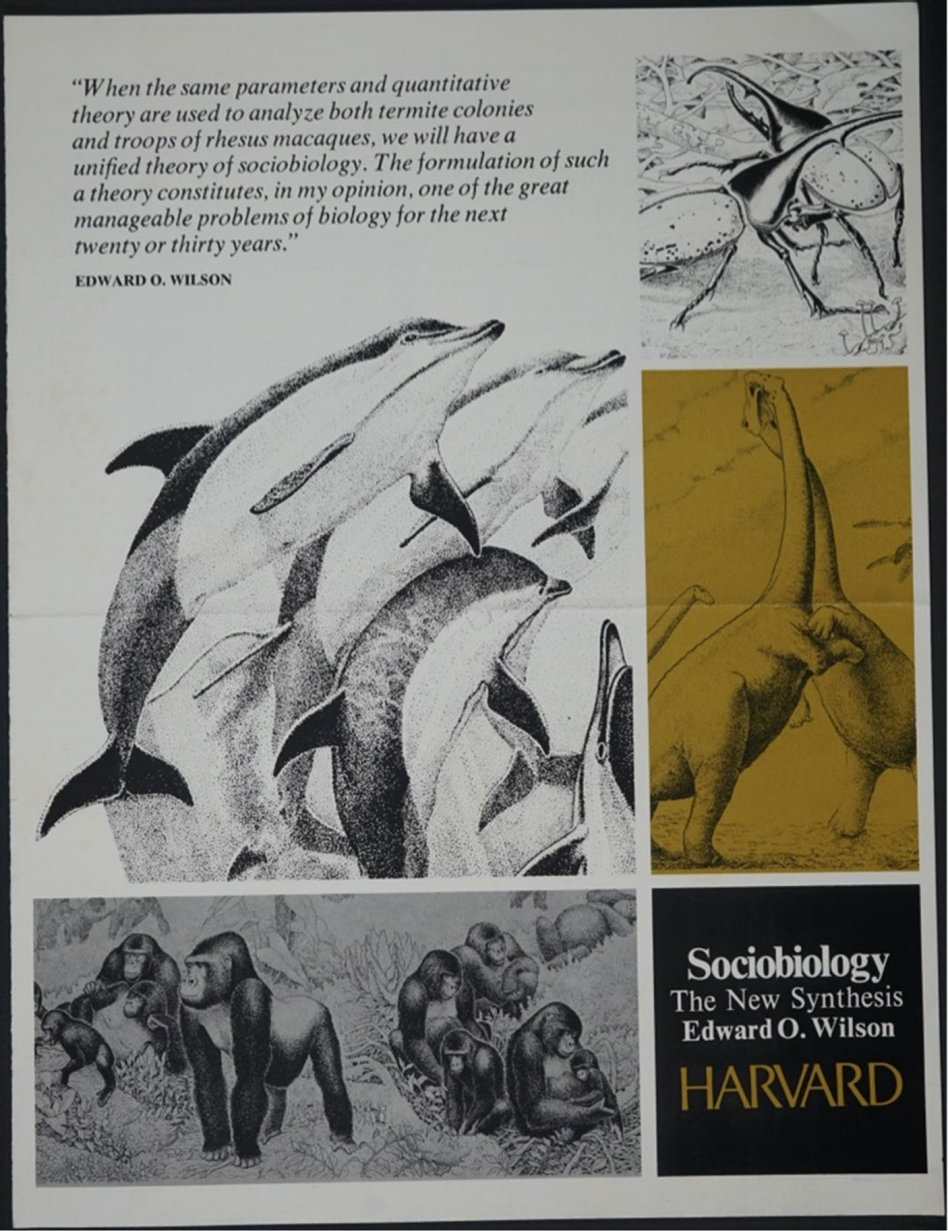
Cover of the promotional brochure for *Sociobiology: The New Synthesis*, ca. 1975. Box 240, EOW Papers.

Since the sweeping aims of the book were unlikely to attract all readers equally, marketing the book to its different intended readerships became central. Therefore, the promotion stressed different aspects for different publics. When positioning *Sociobiology* as a specialist academic book and textbook intended mainly for college courses on animal behavior, promotional material stressed its suitability for teaching and its comprehensive coverage of animal behavior. A draft for the promotional text written by Wilson touted the book as “easily readable by anyone who has had an elementary course in biology at the college level or the equivalent.”^8^ Additionally, he argued that for promotional purposes re-printing the table of contents “would be *most* effective.”^9^ But the promotional text was also addressed to social scientists, emphasizing its intended interdisciplinary impact as “sociobiology is destined to become increasingly fundamental to anthropology, psychology, and sociology.”^10^ The promotional brochure created and distributed by Harvard University Press in 1975 announced: “Now there’s one science for all social creatures,” and unlike the earlier draft framed the understanding of altruism as the “cornerstone of sociobiological theory.”^11^ This also formed the basis for *Sociobiology’s* promotion to the general public: sociobiology as a new science of altruism (Fig. 2).

## “A Science of Altruism” – *Sociobiology* in the Press

Media coverage was essential for *Sociobiology’s* success and in the summer of 1975 press events in distinguished East Coast locations of higher learning were held to introduce Wilson and *Sociobiology* to journalists. One such event took place on May 14, 1975 at the *Museum of Science* in Boston and within two weeks *Sociobiology* made the cover of the *New York Times* on May 28, 1975 under a headline that announced Wilson as “Updating Darwin on Behavior.”^12^ How did Wilson and Harvard University Press manage to streamline almost 600 pages of what was essentially a biology textbook, considered even by academic colleagues to be an “unwieldy tome” (Crook 1976, p. 703), into a message appealing to the public? In studying science and popular culture, Peter Bowler identified two key factors crucial to popular publishing success: an author with an “established public image” and a topic that had social relevance, since the public “needed to be convinced that the book addressed some pressing cultural or social need” (Bowler 2009, pp. 96–98). Press coverage of *Sociobiology* in the summer of 1975 displayed these features perfectly.

A striking example was the first major article about *Sociobiology*, published by science journalist Boyce Rensberger in the *New York Times* about a month before the book’s publication. This article and many that followed focused heavily on Wilson, featuring him through interviews, quoting him, characterizing him as the founder of a new discipline, and creating a public image of an affable, personable, mild-mannered naturalist and an “avid jogger.”^13^ But he was also portrayed as a pillar of scientific integrity: a formidable ant-expert who encompassed expertise, objectivity and measured precision, often by presenting his vision of sociobiology as a moderate option between the extremes of Skinnerian behaviorism and Lorenzian theories of innate aggression. This positive portrayal also resulted from Wilson’s deft personal touch. His interactions with interviewers were patient and polite, with Wilson often sending out cordial thank you notes after meetings or words of approval after articles were published.

Wilson devoted a great deal of time to journalists and was thus also able to shape how sociobiology and its social relevance were portrayed. Since *Sociobiology* was long, complicated, and mainly covered vast amounts of material on animal behavior of dubious popular appeal, Wilson directed journalistic attention to one chapter of the book, the explanation of kin selection as the genetic basis for altruism. A local newspaper vividly described the scene of Wilson highlighting its importance at a promotional event in Woods Hole: “Dr. Wilson, who spent the afternoon looking benevolently through pink-rimmed glasses at a protean circle of admirers, kept stressing that everyone should read Chapter Five of his book. That chapter explains how altruistic behavior ‘makes for the most complicated society’.”^14^ Focusing press attention on this manageable single aspect of the book with strong implications for human social behavior made the event in Wilson’s words “successful beyond all my expectations, drawing an extraordinary group of people and creating an air of excitement over books, publishing, HUP, etc. As for myself personally, in one strike the reception turned a semi-recluse into a local social success.”^15^

The topic of altruism featured prominently in press coverage of sociobiology, especially in the *New York Times*. The article by Rensberger introduced sociobiology as a “nascent discipline” promising the “revolutionary implication that much of man’s behavior toward his fellows, ranging from aggressive impulses to humanitarian inspirations, may be as much a product of evolution as is the structure of the hand or the size of the brain.”^16^ Focusing on the topic of altruism and the genetic basis of its evolution and using Landry’s illustrations, Rensberger introduced *Sociobiology* as a “long awaited definitive book” with its “central theory […] that the social behavior of individuals evolves so as to maximize the chances of genes like the individual’s own to survive in the greatest number.”^17^ Rensberger used examples from animal behavior to present the “self-sacrificing behavior of animals” as an evolutionary puzzle only sociobiological theorizing could solve.^18^ With the Kuhnian language of puzzle-solving, sociobiology was presented as a new paradigm for understanding altruism and *Sociobiology* as the foundational text for this new discipline.

As the summer of 1975 progressed, *Sociobiology* was repeatedly featured as an “Editor’s Choice” and sociobiology itself promoted as a “science of altruism” with a continued focus on its potential contributions to understanding human behavior.^19^ By the end of the year, *Sociobiology* was listed as a “Noteworthy Title,” again highlighting its importance in exploring the biological foundations of altruistic behavior. When Wilson was personally invited to write an article of what he deemed his “best shot at a brief popular account […] of some of the most interesting ideas in my book,”^20^ he once again connected animal and human behavior through the topic of altruism with its headline stating: “Human decency is animal.”^21^ In this article he also positioned sociobiology as a potential guide to societal conflicts by arguing for the acceptance of homosexuality as a natural altruistic behavior.^22^ Focusing on kin selection and altruism as well as the social relevance for human societies led to the streamlined press version of *Sociobiology* evident in Rensberger’s article. Sociobiology was portrayed as a revolutionary discipline based on kin selection that explained animal and human altruism and other social behaviors.

Coverage of this truncated press version of *Sociobiology* as a book about altruism became more sensationalized as its relevance to human behavior and society was more explicitly addressed. The example of Rensberger’s article is especially instructive; it did not only appear in the *New York Times*, but was also reprinted in dozens of other newspapers under headlines such as “How we act may be in our genes” or “Behavior patterns linked to evolution,” emphasizing aspects such as genes or evolution influencing human behavior. Other headlines suggested that sociobiology was a direct challenge to the social sciences and included “Theory shakes social sciences,” or more broadly “This scientist could shock you,” next to a picture of Wilson.^23^ In the summer of 1975, more articles appeared that stressed sociobiology’s social relevance for humanity.

The alternative Boston area newspaper *The Real Paper* praised Wilson for his nuanced perspective on human behavior because he “states explicitly that human behavior is highly plastic, that it represents a complex response to a complex environment.”^24^ In this article Wilson is quoted as hoping “that by discovering the history of the human mind, sociobiology will reveal the full range of behavior of which man is capable – and more than that, the evolutionary rationale behind that range.”^25^ This discovery might also have applications as Wilson “suggests quite tentatively that this knowledge would allow us to select which kinds of social behavior we would like to encourage.”^26^ An article in the *Boston Globe* explained which social behaviors Wilson had in mind, which were “restraint in population growth and use of resources and more cooperative behavior” as a response to overpopulation, depletion of natural resources and the dangers of nuclear annihilation.^27^ Sociobiology’s social relevance, Wilson advocated, lay in its capacity to provide scientific solutions to current threats humanity faced, to “offer some guidance and warning signs in this transition phase” toward an ecological steady state of “zero population growth, continuous recycling of all resources and ‘a pretty tightly controlled and regimented physical world.’”^28^ In addition to providing knowledge about where humanity has been, sociobiology became a discipline useful for shaping the future by supplanting knowledge from the social sciences and reassessing ethical questions and moral dilemmas through an evolutionary perspective.

In only a few weeks after its publication *Sociobiology* had an immense exposure. Some twenty-five articles about S*ociobiology* appeared in newspapers across the United States between May 28 and August 16, 1975. Wilson graciously thanked the journalists from the *Real Paper* and the *Boston Globe* for their articles.^29^ But he paid a special compliment to Rensberger, whose *New York Times* article was reprinted at least fourteen times, for “creating a favorable stir,” remarking that: “I’ve discovered that one of the few things that can make Harvard professors aware of the existence of another Harvard professor is a story on the front page of the N.Y. Times!”^30^ At its core, press promotion of sociobiology presented an optimistic view of human nature and scientific progress. It suggested that humanity could move toward a future where social and ecological problems were permanently resolved. The press campaign was successful in promoting *Sociobiology* as containing key insights into complex human societies based on evolutionary theory. But doing so not only misrepresented the tone and content of most of the book, it also advertised its appeal with claims from the section of the book that was unanimously seen as weakest by specialist reviewers.

## “One Science for all Social Creatures”? – The Limits of *Sociobiology’s* Ambition

**Fig. 3:**
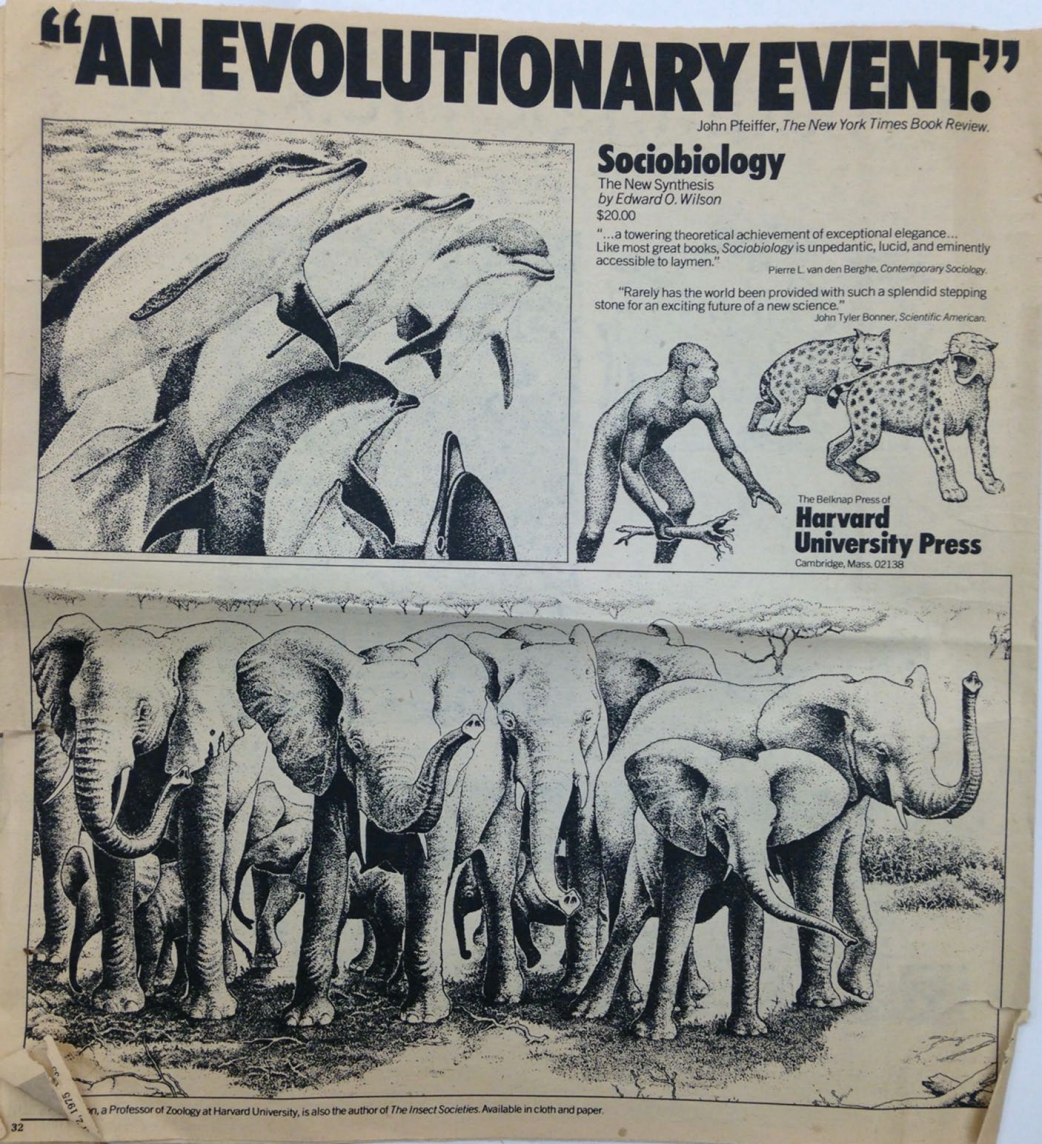
Newspaper clipping of the print advertisement for *Sociobiology: The New Synthesis* that was printed in *New York Times Book Review*, November 2, 1975, p. 34. Box 240, EOW Papers.

*Sociobiology* was everywhere. But the promotion did not rely on press coverage alone. Harvard University Press had compiled a five-page list of possible reviewers in popular and scientific journals and generously distributed review copies. This strategy also yielded quick success as *Sociobiology* was reviewed in a slew of popular science journals and the two most important general interest review papers at the time: The *New York Review of Books* and the *New York Times Book Review.* Only a select few scholarly monographs, “the elite of the elite” (Lindholm-Romantschuck 1998, p. 72), managed to secure reviews in both outlets, which did not only signal academic prestige but also demonstrably impacted sales.^31^ Satisfied, Wilson noted in handwriting on the *New York Times Book Review* piece by John Pfeiffer: “And more! This is now on its way to become the most publicized book on science in years.”^32^ Indeed, *Sociobiology* was extremely widely reviewed in journals that bridged scientific, literary and educated audiences. In short order, reviews also appeared in prestigious semi-popular scientific magazines and journals such as *Natural History Magazine*,* Harper’s Magazine*,* Scientific American* and the *New Scientist.* Given the wide readership of these magazines, the reviews written by eminent biologists did not serve as mere critiques or descriptions of the book but also effectively functioned as part of the promotional campaign (Fig. 3).

This is most apparent in the review of Wilson’s *Sociobiology* by Pfeiffer, himself a science popularizer with a background in anthropology and author of *The Emergence of Man* (1969). Pfeiffer concluded his reviews stating that the book “has much to say about us here and now,” especially when it came to cooperation and altruism and closed with the line that *Sociobiology* should be regarded as “an evolutionary event in itself announcing for all who can hear that we are on the verge of breakthroughs in the effort to understand our place in the scheme of things.”^33^ This provided Harvard University Press with an ideal new slogan. The original tagline, “[n]ow there’s one science for all social creatures,” used in print advertisements and the promotional brochure,^34^ was replaced with Pfeiffer’s quotation and hailed *Sociobiology* as “An Evolutionary Event.” Other enthusiastic endorsements for the print advertisements were taken from reviews by biologist John Tyler Bonner and sociologist Pierre van den Berghe. They matched the promotional agenda of the press: “The three quotes we are using balance beautifully in copy and also in interest appeal (one popular, one behavioral, one scientific).”^35^ The mood in the letters between Harvard University Press and Wilson in late October 1975 was self-congratulatory with the press calling the book a “show stopper,” and musing that “the Press’s worst problem will be deciding which superlatives to use!”^36^

Superlatives were extensively employed in reviews for *Sociobiology*. But even in more popular outlets, reviewers took their evaluative function seriously and dissected the different ambitions of the book. In their assessment it had undoubtedly succeeded in its original intent of being a compendium of animal behavior theory and empirical data. But the promotion had raised expectations that *Sociobiology* was also going to address the topic of human behavior with nuance and insight. Closer examination of these reviews, however, reveals that despite an overall positive assessment of the book, *Sociobiology* failed on this account.

Almost all reviewers were laudatory regarding the ambition and scope of the book and marveled at Wilson’s ability to synthesize a huge amount of animal behavior studies in a clear and concise manner. Developmental biologist Conrad H. Waddington stressed that Wilson was “astonishingly successful in achieving” his “extraordinarily ambitious aim” in the *New York Review of Books,* and wrote that *Sociobiology* “will undoubtedly be for many years to come a major source of information about all aspects of our knowledge of social behavior in animals.”^37^ Cancer researcher and scientific essayist Lewis Thomas writing for *Harper’s Magazine* described the reading experience as “an ocean of information, in which facts are adrift like plankton” and lauded Wilson’s “uncompromised scholarship.”^38^ A similar consensus was reached in popular science magazines. Former Director of the Biological Sciences at Cornell University Robert S. Morison writing for *Natural History Magazine* was so impressed that his review began with a disclaimer that “it is highly probable that the only person qualified to review this book is the author himself,” and continued to call *Sociobiology* a “tour de force of collecting, winnowing, interpreting, speculating, and publishing evidence for a biological explanation of behavior.”^39^ Writing for *Scientific American*, evolutionary and developmental biologist John Tyler Bonner called it an “elegant compendium of all the significant things we now know about animal societies.”^40^ The consensus was that *Sociobiology* did indeed achieve a synthesis, at least “regarding animal behavior: it stands alone, almost certainly a landmark, quite possibly a monumental one.”^41^

But Wilson’s ambition of synthesizing the behavior of all social creatures hit a limit with the human chapter. Just as much as reviews converged on its achievement regarding animal behavior, they also converged on its flawed, limited or misleading treatment of human behavior. Even the reviews that were considered promotional by Wilson and Harvard University Press took a dimmer view of the book’s final chapter on humans. Pfeiffer, for example, put it succinctly: “Wilson falters somewhat in presenting the human story.”^42^ He was especially skeptical of Wilson’s uncritical adoption of the already disputed theory that cooperative hunting of big game was central to human evolution and felt Wilson more generally underestimated the profound influence of culture, which had increasingly driven human development alongside biology. Waddington saw similar shortcomings. As the review title “Mindless Societies” emphasized, in Waddington’s estimation the jump from natural selection straight to social behavior left a huge analytical gap, which he called “the weakest feature in the whole grand structure which Wilson has built up.” He asked: “Is it not surprising that in a book of 700 large pages about social behavior there is no explicit mention whatever of mentality?”^43^ This lack of any conceptualization for “mind, mentality, purpose, goal, aim, or any word of similar connotation” in the entire book,^44^ was already a problem for Waddington when it came to animals. But in the human case this was furthered by Wilson’s neglect of the social transmission of knowledge and the many insights gleaned from cultural anthropology, for example by scholars such as Margaret Mead. Culture and mind were given short shrift, in other words. This criticism extended to Wilson’s claim about the necessity of an evolutionary ethics. Evolutionary theorist John Maynard Smith forcefully disagreed in his review for the *New Scientist* that understanding the evolutionary roots of conscience (though he agreed it probably had such roots) would tell us anything relevant about how to settle moral issues.^45^

Writing for general interest or popular science magazines did not result in uncritical reviews. Most reviewers were apprehensive about the last chapter and some of them very explicit in their disagreement with Wilson. They warned their readers that *Sociobiology* had nothing scientifically valid to say about humans as it was underestimating the complexities of mind, culture and ethics. The book fell short of its promotional promise to the public.

## “A Patchwork Without a Bold New Theoretical Pattern of its Own” – Reviews in Biological Journals

The promotional campaign had also created the expectation that *Sociobiology* was more than a compendium on animal behavior, but the foundational text of a new biological discipline that integrated different biological fields based on evolutionary theory. The assessment of *Sociobiology* as “a splendid stepping stone into the future of a new science” from Bonner’s review for the *Scientific American* may have sounded like whole-hearted approval to the Harvard University Press office.^46^ But it actually expressed an apprehension many biologists shared. In their reviews, the consensus emerged that Wilson oversold his theoretical contributions and was premature in declaring sociobiology a discipline.

Curiously enough, some opposition was sparked by Wilson’s reliance on altruism as sociobiology’s theoretical cornerstone. Waddington argued this emphasis on altruism was “most obscure” and saw communication as the obvious central issue, implying that Wilson mischaracterized his own work.^47^ While Waddington at least appreciated that Wilson did not claim that kin selection was the only explanation for the emergence of altruism, this theoretical openness in turn alienated other evolutionary theorists. To them Wilson’s account of kin selection read as muddled and misleading. Maynard Smith praised Wilson’s “attitude to theory” as “admirable” in the *New Scientist*,^48^ but in a letter to Wilson expressed his concerns with placing kin selection on a continuous spectrum with group selection.^49^ In his response Wilson readily conceded his mistakes: “I regret that I didn’t see it more clearly when I wrote the book,”^50^ he wrote, and revealed that Robert Trivers, at the time an assistant professor at the biology department at Harvard, had already corrected him on this issue. This background helps explain why, in his review, Maynard Smith sought to set the record straight by detailing his own development of “kin selection” and highlighting William Hamilton’s “decisive step” in his formulation of inclusive fitness.^51^

Hamilton himself later bemoaned the lack of evolutionary game theory, a contribution by Maynard Smith, and summarized Wilson’s theoretical acumen as sailing with tangled rigging “without stars if need be, hitting islands by some sort of dead reckoning and the look of the sea” (Hamilton 1977, p. 975). Unlike what the press promotion suggested, Wilson’s presentation of the theory on altruism was a tangled mess. This theoretical weakness formed part of a larger pattern many reviewers identified: Wilson’s use of theory and models did not quite coalesce into a coherent framework. Waddington had already described Wilson as “too impressed by certain theories,”^52^ and primatologist Donald Stone Sade noted that Wilson’s models had not been applied to natural populations and remained “working hypotheses” (Sade 1975, p. 262). In a later review evolutionary biologist Mary Jane West-Eberhard characterized *Sociobiology* “as a patchwork neatly stitched from relevant pieces of other fields, without a bold new theoretical pattern of its own” (West Eberhard 1976, p. 92) Even the most sympathetic reviewers mentioned that Wilson’s “eagerness to tie things together” led to generalizations that “may not withstand scrutiny” (Barash 1976, p. 399).

But most biologists found value in the preliminary synthesis Wilson had provided. Some, like the slime mold specialist Bonner, or David Barash who worked on ecological factors of marmot behavior, appreciated that Wilson aimed to bring population biology and ecology into closer proximity on the topic of social behavior. Bonner even identified the search for ecological correlates of sociality as one basis of sociobiology, its central question being: “What environmental conditions have promoted a social existence?”^53^ Bonner’s assessment seems to have been right on the mark. Wilson thanked him for “clearly explaining what the book is about – something no other reviewer has managed to do.“^54^ For biologists like Bonner it was no contradiction to conclude that this was “the least developed of all the foundations of sociobiology, and perhaps for that reason it is one of the most interesting”^55^

Equally, they did not mind the more speculative style of Wilson’s synthesis. In their assessment even speculative extrapolations remained firmly in the realm of science, as it was not necessarily the accuracy of the content, but “the consistent display of scientific reasoning that insists that theory in all its aspects must be subjected to testing and potential falsification” that made *Sociobiology* fulfill the standards of science (Sade 1975, p. 262). The “most insidious weakness of sociobiological theory” was that it was easier to create models than to generate the empirical data required to test them. But this could actually be turned into a strength for field biologists since conducting sociobiological research would “require revision of the goals of fieldwork and a reallocation of resources for genuine long-term research” (Sade 1975, pp. 262–263). Field biologists could argue that more funding and recognition should be given to their work since it formed the empirical basis for the proposed unified discipline with implications for human social behavior.

The reviews reveal a sense of excitement and energetic confidence that sociobiology offered a glimpse into future directions of studies on animal behavior. And as they did not expect *Sociobiology* to present a contained and finalized synthesis, they were neither disappointed nor irritated that rather than an ending, it represented the beginning of a “period of profitable confusion and theoretical indigestion” (Sade 1975, p. 262). They praised Wilson’s book for its willingness to explore the potential of sociobiological explanations and were in turn willing to overlook that in some aspects this exploration was scientifically incomplete, incorrect, or preliminary. Biologists reviewing the book did not judge Wilson’s *Sociobiology* to be unscientific as a whole, but this did not commit them to granting sociobiology the disciplinary status Wilson had aspired to. They saw sociobiology as the beginning of a new field “that undoubtedly will hold a central position in biology, and perhaps in sociology too, for many years to come” but expressed doubts about whether it made sense to declare it a discipline.^56^

This assessment became especially visible in the multiple reviews published in *Animal Behavior*. Such review rounds were done for important books in the field and included an *Author’s Précis* and *Author’s Reply* section. Patrick Bateson, a central figure in the study of animal behavior at Cambridge University, wrote to Wilson in September 1975 that *Sociobiology* was “just the kind of book for which the journal should publish a multiple set of reviews.”^57^ Published in August 1976, animal behaviorists, mainly from the United Kingdom and Europe, showed themselves to be impressed by Wilson’s thematic synthesis but pointed to similar weaknesses as the earlier reviewers. They were mostly skeptical about announcing sociobiology *as a discipline*, even when limited to animal behavior. In positioning sociobiology as a new discipline, Wilson had portrayed ethology as broadly unscientific and ripe for “cannibalization” (Wilson 1975, p. 6). This demarcation alienated some animal behaviorists and they stressed the continuity with previous developments in ethology, suggesting that social ethology and behavioral ecology were more appropriate disciplinary vessels for the contents of sociobiology. Leading animal behaviorists Robert Hinde and John Crook accordingly discounted the proclamation of sociobiology as a discipline as either a “political game” (Crook 1976, p. 703) or “sales talk” (Hinde 1976, p. 707). In the end, there were not many adherents to Wilson’s disciplinary ambitions;^58^ much more empirical work needed to be done and as Crook concluded “whether we eventually call this sociobiology, evolutionary ethology or biosociology really does not matter” (Crook 1976, p. 703).

Instead of unifying different strands of animal behavior research, *Sociobiology* amounted to an individualistic effort that aimed to supersede ongoing developments in the field. Wilson acknowledged both the preliminary status and particular perspective of *his* sociobiology when he wrote in his *Author’s Response* that *“*a more accurate title of the book might have been Sociobiology: Toward a Partial Synthesis” (Wilson 1976, p. 717). Wilson with his disciplinary ambition neither provided the theoretical cohesion nor inspired the social unity necessary for the foundation of a discipline.

## “A Stimulating Prodding of the Sociological Imagination” – Reviews in Sociological Journals

Despite biologists harboring doubts about sociobiology’s disciplinary status within biology, they took Wilson’s ambition to bring social scientists into the evolutionary fold seriously. It was even declared to be the real test of sociobiology’s viability as a discipline, since “to be complete, sociobiological theory must account for the emergence of man and absorb both anthropology and sociology” (Sade 1975, p. 263). But biologists were unsure if Wilson’s approach was inviting and his content enticing enough to attract social scientists. Morison wondered if announcing sociology’s integration into the “Modern Synthesis” was “the best way of luring disciples of these historic schools to read the book.” Or was Wilson’s disciplinary boldness more like a pool player unwisely “calling one’s shots in advance”?^59^ And even if social scientists did read it, would Wilson’s summary of what biology offered be “a perspective that [they] will find valuable and useful?”^60^ Biologists who reviewed the book recognized how important the reaction of social scientists would be for Wilson’s broad disciplinary project; and most expected *Sociobiology* to be a hard sell. They predicted a “trial-by-fire […] with the formidable complexity of human society” (West Eberhard 1976, p. 91), or warned that Wilson could turn into a “Saint Sebastian of sociobiology” with the “protagonists of the conventional wisdom in economics, sociology, psychology and indeed biology itself […] even now aiming their arrows in his direction.”^61^

Biological approaches were at the margins of sociology, and it is unlikely that the book would have garnered huge interest without first its promotion and later the controversy surrounding it. Most sociological reviews were written after the initial controversy, and both *The American Journal of Sociology* and *Contemporary Sociology* bundled several reviews with different perspectives on the book. Most reviewers were part of a small community of sociologists that had already engaged with biological explanations and were open to them. Consequently, reviewers with a biosocial perspective like Pierre van den Berghe, whose quotation was used for the advertisement, concluded *Sociobiology* “will make further resistance to biological concepts not only difficult but patently silly” (Van den Berghe 1976, p. 731). Others were less enthusiastic but still welcomed Wilson’s contribution of bringing “visibility and credibility to legitimate the biological approach” (Mazur 1976, p. 700). But even for those sociologists open to biological approaches, Wilson’s book fell short of its ambitions. An example of this was the review by Gerhard Lenski in *Social Forces*. Like most reviewers Lenski had a generally positive impression of Wilson’s stated intention of bringing biology and sociology into closer contact and defended him against accusations of determinism. This, however, did not mean embracing what he wrote about human behavior and sociology. For those unwilling to engage with cross-species comparative studies of social behavior, Wilson and his *Sociobiology* even offered a “convenient excuse in his discussions of sociology and of human societies” (Lenski 1976, p. 530).

What troubled reviewers was Wilson’s surface-level understanding of sociology, “gross generalizations about the field” and the “disappointing” chapter about humans (Mazur 1976, p. 697). The disappointment began with the title which some reviewers initially understood as a synthesis between sociology and biology but soon found that Wilson engaged little with sociology (Tiryakian 1976, p. 701). Wilson had relied mostly on the introductory textbook by Gerhard and Jean Lenski and his “selective and biased” (Eckland 1976, p. 695) readings of Parsons, Levi-Strauss, Weber, and Durkheim led him to regard sociology as an “essentially nontheoretical, descriptive science” (Lenski 1976, p. 530). Because sociology offered many theories, Wilson concluded, it had none to be taken seriously. Wilson perceived sociology’s theoretical diversity as an issue to be solved by introducing evolutionary theory as a new basis for the social sciences which would then unify the social sciences theoretically and include them in the “Modern Synthesis.” But even for sociologists open to importing biological theory, there were doubts about what theoretical framework sociobiology could even provide. Far from being overwhelmed by sociobiology’s scientism, it was Wilson’s sociobiology which lacked scientific rigor. Its theoretical underpinning was not suited for an empirical approach, as it was deemed too tautological, too vague or untestable (Mazur 1976, p. 698/9). In addition to his failure to appreciate the theoretical variety of the discipline, his understanding of sociology also completely neglected empirical sociological studies. Therefore, sociologists felt rather underestimated by Wilson methodically, stating that their discipline was more statistically sophisticated than the new discipline Wilson promoted (Eckland 1976, p. 696). Sociobiology in its current form thus addressed neither theoretical nor empirical needs of sociologists.

For Lenski, Wilson was only the latest iteration of the proselytizers of the “Modern Synthesis” who tried to expand into sociology. He read Wilson’s disciplinary ambition rather charitably as an invitation to collaboration rather than “intellectual imperialism” and wanted “to open the channels to serious and continuing intellectual dialogue” to counter the reflexive reductionism of biologists (Lenski 1976, p. 530). Maybe, he concluded, sociology could be included in the Modern Synthesis, but only if sociological concepts and interests were in turn integrated in a future collaboration between the disciplines. Disciplinary unification was not a one-way street.

Others were more explicit in their rejection of disciplinary reductionism as impossible because there was an “almost incomprehensible causal distance between gene action and phenotypic behavior in man” (Eckland 1976, p. 696). Rather than the promise of unification for sociologists with no biological leanings, Sociobiology invited merely “cautious, limited convergence” (Eckland 1976, p. 697). They showed an interest in evolutionary thought and curiously explored the chapters on animal social mechanisms which read “almost like text in sociology or anthropology” (Eckland 1976, p. 694), but there was little knowledge to be gleaned about human societies from the book and it was not of much use for sociological research. Still, they wanted to take a middle ground between “naive ignorance, a polemical rejection, or a docile acceptance.” To them the best use of sociobiology was as a “stimulating prodding of the sociological imagination” (Tiryakian 1976, p. 705).

Most reviewers did not see the need to pick up their bow and arrow but expressed pragmatic and methodical concerns about *Sociobiology’s* utility for sociologists. Reviewers with a more biological outlook hoped optimistically for a “coming to terms with Neo-Darwinian synthesis” (Blute 1976, p. 731), but braced for another round of the nature-nurture debate. While most sociologists expressed a wish to move beyond a false nature-nurture dichotomy and toward more productive collaboration with biologists, *Sociobiology* was not the right tool for the job. They instead recommended the less speculative works by Robert Hinde as “more useful for sociologists” (Mazur 1976, p. 700). *Sociobiology* did not provide enough of a theoretical or methodological foundation for its unifying ambition.

## “An Infant Discipline” – *Sociobiology’s* Disciplinary Ambiguity

Wilson opened the chapter about the “Sociobiology Controversy” in his 1994 autobiography with a reflection on the reviews it garnered. He recalled that reviews “whipsawed it with alternating praise and condemnation” and immediately dichotomized the reaction along disciplinary lines with biologists being “almost unanimously favorable” and social scientists rejecting the book (Wilson 2006, p. 330). The ensuing debates must have clouded his memory. Most reviews, regardless of disciplinary affiliation, identified the same strengths and weaknesses of *Sociobiology*. Later reviews in specialized sociology journals tended to praise and criticize *Sociobiology* along similar lines of the earlier reviews written by biologists even before the controversy erupted. The scientific reception of the book did decisively not mirror the categories of “for” or “against” that were so definitively constructed in public. Instead, the reception of the book converged on certain points: its immense scope as a compendium for animal behavior, its vagueness and inaccuracies regarding human behavior and its limited utility as a foundational text for a discipline, but at best a starting point to pursue these disciplinary ambitions further.

Ruminating on the reasons for the controversy in his autobiography Wilson concluded that the “hybrid nature” of the book, covering both humans and animals, was a major factor: “The human chapters were rendered creditable by the massive animal documentation, while the biology gained added significance from the human implications” (Wilson 2006, p. 336). But this was only true in the public sphere due to the promotional campaign in the press. Where the press condensed *Sociobiology* with Wilson’s assistance to one central theory, disciplinary reviewers perceived an eclectic combination of theories and models. Where the press touted new explanations for certain behaviors, reviewers noted lacking empirical data regarding animals and speculations regarding humans. Where the press coverage had praised *Sociobiology* as containing a new discipline with the implications of revolutionary insights and the latest discoveries, the reviewers agreed that *Sociobiology* remained speculative and preliminary. Specialist reviewers clearly separated the two books as Wilson had originally intended them and even those sympathetic to his project concluded “that sociobiology is still too immature a scientific endeavor to be presented to public scrutiny as a *fait accompli*. For his boldness, perhaps arrogance, Wilson deserves some of the denunciations he has received” (Bates and Lees 1976, p. 355).

The harshest of these denunciations were epitomized by the critical letter by the Sociobiology Study Group in response to Waddington’s review in the *New York Review of Books.* In the letter the critics spoke specifically to this public construction of sociobiology as a disciplinary *fait accompli* and aimed to deconstruct *Sociobiology’s* scientific status in the public sphere, a status that resulted from its press campaign. The main goal of the letter was to identify “strategies and sleights of hand“ meant to persuade the public of its scientific validity.^62^ In a point by point analysis these strategies were highlighted using concrete examples from the book; they included employing metaphors that blurred the boundary between humans and animals, simplistic talk of genes for behavior, reconstructing an evolutionary pre-history that builds on prejudice and stereotypes, and, finally, inventing ad hoc arguments and effects that amount to mere speculation. The main function of the letter was not to provide a comprehensive scientific critique, but to educate the public and encourage more discernment regarding Wilson’s “alleged creation of a new discipline.”^63^ It was specifically the combination of weak scientific basis for its human chapter and grandiose public promotion that made *Sociobiology* politically and socially dangerous.

Most biologists agreed that Wilson’s project of laying the foundation for sociobiology by the power of his “golden pen” (West Eberhard 1976, p. 89) was far from completed. However, unlike the Sociobiology Study Group they regarded it as a project with potential as sociobiology was often described as a discipline “in its infancy.”^64^ This conveyed the sense that sociobiology, like an infant, required proper care and patience so that it might be nurtured into a mature science. Wilson himself acknowledged freely in the book and elsewhere that sociobiology was a “relatively young discipline, the boundaries of which have been newly defined by a compositionist or synthetic approach, in the time-honored procedure of evolutionary biology” (Wilson 1976, p. 716). But precisely his attempt of defining the discipline through his own synthetic monograph raised a central issue for its future development. It presented Wilson’s idiosyncratic and often erratic understanding of the developing discipline as central and its main starting point and in doing so sidelined similar undertakings. But non-Wilsonian endeavors continued to develop largely without considering *Sociobiology* as a relevant starting point; contrary to Wilson’s later claims the researchers in these fields had more reasons to avoid the term sociobiology than the wish to avoid political controversy.

*Sociobiology* struggled to breathe under the weight of serving multiple competing ambitions all in one. “It is difficult for a single book to be all things to all people,“ concluded Mary Jane West-Eberhard, “But how much can one ask of one book, and one man, at one time?” (West Eberhard 1976, p. 92). The appeal to different publics was characterized as one reason for its shortcomings and it was doubtful “whether the format chosen for this book is sufficiently lucid and penetrating to convey the essence of its contents to a widely differentiated readership” (Baerends 1976, p. 699). But through examining the promotion and reception of *Sociobiology* we see the difficulty of speaking of its essence in the first place. The term “sociobiology” already carried multiple disciplinary meanings at the time of its publication. Through the conception and promotion of *Sociobiology* as well as its early reception more were added. Over the course of the debates surrounding it, many more were accumulated, while others were discarded. Its meaning shifted and transformed in the fifty years since the publication. But already in 1975 this single book was many things to different people. Its legacy remains ambiguous.

## Footnotes

1 This meaning focuses on Wilson’s written words, and may also cover his other book-length efforts to establish a discipline that encompasses both animal and human social behavior, such as *On Human Nature* (1978) which he presents as an extension of sociobiology, or his later works written with Charles Lumsden such as *Genes Mind and Culture* (1981) and its more popular version *Promethean Fire* (1983), which responded to the criticism that early sociobiology failed to consider the role of mentality and cultural transmission in humans. This shows that even limiting the meaning to one author’s understanding and a short time frame of eight years, entails significant shifts in what the term sociobiology can cover.

2 Since 1997 this journal has been known as *Human Behavior and Evolution*.

3 Nina McCain, “Sociobiology: New theory on man’s motivation,” *Boston Globe*, July 13, 1975, p. A4.

4 Despite Wilson’s assertion that “most of it can be read with full understanding by any intelligent person” without a scientific background, reviews at the time repeatedly stated that sections of the book were too technical or advised skipping entire sections (Wilson 1975, p. 577).

5 Wilson to Arthur Rosenthal (HUP), June 5, 1973, Box 87, Edward O. Wilson Papers, Manuscript Division, Library of Congress, Washington D.C. (hereafter cited as EOW Papers) and Wilson to Arthur Rosenthal (HUP), April 29, 1974, Box 87, EOW Papers.

6 Wilson to Margaret Ann Roth (HUP), May 28, 1974, Box 87, EOW Papers.

7 E.O. Wilson, “The prospect for a unified sociobiology,” *American Scientist*, July–August 1971, p. 400–401.

8 Wilson to Margaret Ann Roth (HUP), May 28, 1974, Box 87, EOW Papers.

9 Emphasis in original, Wilson to Margaret Ann Roth (HUP), May 28, 1974, Box 87, EOW Papers.

10 Wilson to Margaret Ann Roth (HUP), May 28, 1974, Box 87, EOW Papers.

11 Promotional Flyer, undated, Box 240, EOW Papers.

12 Boyce Rensberger, “Updating Darwin on behavior,” *New York Times*, May 28, 1975.

13 Gail Jennes, “Sociobiology is a new science with new ideas on why we sometimes behave like cavemen,” *People Weekly*, November 17, 1975, p. 69.

14 Dixie Brown, “Harvard Univ. Press hosts a Woods Hole happening,” *Falmouth Times*, July 25, 1975.

15 Wilson to Arthur Rosenthal (HUP), July 23, 1975, Box 87, EOW Papers.

16 Boyce Rensberger, “Updating Darwin on behavior,” *New York Times*, May 28, 1975, p. 1.

17 Boyce Rensberger, “Updating Darwin on behavior,” *New York Times*, May 28, 1975, p. 52.

18 Boyce Rensberger, “Updating Darwin on behavior,” *New York Times*, May 28, 1975, p. 52.

19 “Sociobiology, a science of altruism,” *New York Times*, June 1, 1975.

20 Wilson to Howard Musin (New York Times), June 24, 1975, Box 126, EOW Papers.

21 E.O. Wilson, “Human decency is animal,” *New York Times Magazine*, October 12, 1975.

22 This article was later used by Wilson to counter the charge of biological determinism as he explicitly warned against committing the “naturalistic fallacy” as a “dangerous trap which can only be avoided by constant vigilance.” He wrote “When any genetic bias is demonstrated, it cannot be used to justify a continuing practice in present and future societies. Since most of us live in a radically new environment of our own making, the pursuit of such a practice would be bad biology; and like all bad biology, it would invite disaster” (E.O. Wilson, “Human decency is animal,” *New York Times Magazine*, October 12, 1975, p. 50).

23 See “How we act may be in our genes,” *Detroit Free Press*, June 1, 1975; “Sociobiology: Behavior patterns linked to evolution,” *Tampa Tribune*, June 25, 1975; “This Scientist Could Shock You,” *The Evening Sun* (Baltimore), June 11, 1975; “Theory shakes social sciences,” *The Morning News* (Wilmore, Delaware), August 27, 1975.

24 Rid Brown, “Blue Genes,” *The Real Paper*, July 23, 1975, p. 8.

25 Rid Brown, “Blue Genes,” *The Real Paper*, July 23, 1975, p. 8.

26 Rid Brown, “Blue Genes,” *The Real Paper*, July 23, 1975, p. 8.

27 Nina McCain, “Sociobiology: New theory on man’s motivation,” *Boston Globe*, July 13, 1975, p. A4.

28 Nina McCain, “Sociobiology: New theory on man’s motivation,” *Boston Globe*, July 13, 1975, p. A4.

29 Wilson to Nina McCain, July 14, 1975, Box 25, EOW Papers; Wilson to Rid Brown, July 23, 1975, Box 156, EOW Papers.

30 Wilson to Boyce Rensberger, June 2, 1975, Box 126, EOW Papers.

31 By late November 1975 around 14.000 copies were sold, Diagram of sale numbers, December 2, 1975, Box 240 EOW Papers.

32 Handwritten note on newspaper clipping of Pfeiffer’s review, Box 261, EOW Papers.

33 John Pfeiffer, Review of *Sociobiology. The New Synthesis*, by E.O. Wilson. *New York Times Book Review*, July 27, 1975, p. 15. This quotation was later cited in the letter by the Sociobiology Study Group as part of what they described as a “wave of publicity and laudatory reviews“ (Elizabeth Allen, Barbara Beckwith, Jon Beckwith, Steven Chorover, David Culver, Margaret Duncan, Steven Gould, Ruth Hubbard, Hiroshi Inouye, Anthony Leeds, Richard Lewontin, Chuck Madansky, Larry Miller, Reed Pyeritz, Miriam Rosenthal and Herb Schreier, “Against Sociobiology,” *New York Review of Books*, November 13, 1975, p. 43).

34 Advertisement for *Sociobiology*, July 27, 1975, p. E6. It was also printed in the July/August 1975 issue of the journal *Society*, p. 86.

35 Margaret Ann Roth (HUP) to Wilson, October 28, 1975, Box 87, EOW Papers.

36 Margaret Ann Roth (HUP) to Wilson, October 28, 1975, Box 87, EOW Papers.

37 Conrad H. Waddington, “Mindless societies,” *New York Review of Books*, August 7, 1975, p. 31.

38 Lewis Thomas, “Survival by self-sacrifice,” *Harper’s Magazine*, November 1975, p. 99 and 101.

39 Robert S. Morison, “The biology of behavior,” *Natural History Magazine*, November 1975, p. 86 and 85.

40 John Tyler Bonner, “A new synthesis of the principles that underlie all animal societies,” *Scientific American*, October 1975, p. 132.

41 Robert S. Morison, “The biology of behavior,” *Natural History Magazine*, November 1975, p. 87.

42 John Pfeiffer, Review of *Sociobiology. The New Synthesis*, by E.O. Wilson. *New York Times Book Review*, July 27, 1975, p. 15.

43 Conrad H. Waddington, Mindless societies. *New York Review of Books*, August 7, 1975, p. 31.

44 Conrad H. Waddington, Mindless societies. *New York Review of Books*, August 7, 1975, p. 31.

45 John Maynard Smith, “Survival through suicide,” *The New Scientist*, August 28, 1975, p. 496.

46 John Tyler Bonner, “A new synthesis of the principles that underlie all animal societies,” *Scientific American*, October 1975, p. 132.

47 Conrad H. Waddington, Mindless societies. *New York Review of Books*, August 7, 1975, p. 31.

48 John Maynard Smith, “Survival through suicide,” *The New Scientist*, August 28, 1975, p. 496.

49 John Maynard Smith to Wilson, July 2, 1975, Box 116, EOW Papers.

50 Wilson to John Maynard Smith, July 17, 1975, Box 116, EOW Papers.

51 John Maynard Smith, “Survival through suicide,” *The New Scientist*, August 28, 1975, p. 496.

52 Conrad H. Waddington, Mindless societies. *New York Review of Books*, August 7, 1975, p. 31.

53 John Tyler Bonner, “A new synthesis of the principles that underlie all animal societies,” *Scientific American*, October 1975, p. 130.

54 Wilson to John Tyler Bonner, October 15, 1975, Box 24, EOW Papers.

55 John Tyler Bonner, “A new synthesis of the principles that underlie all animal societies,” *Scientific American*, October 1975, p. 130.

56 John Tyler Bonner, “A new synthesis of the principles that underlie all animal societies,” *Scientific American*, October 1975, p. 129.

57 Patrick Bateson to Wilson, September 22, 1975, Box 237, EOW Papers.

58 When *Sociobiology* was voted the most important book on animal behavior of all time as Wilson and later editions of *Sociobiology’s* dust jacket boast, it was likely in recognition of his synthesis of animal behavior, rather than a vindication for Wilson’s declaration of a new discipline, or his questionable forays into human behavior.

59 Robert S. Morison, “The biology of behavior,” *Natural History Magazine*, November 1975, p. 87.

60 John Tyler Bonner, “A new synthesis of the principles that underlie all animal societies,” *Scientific American*, October 1975, p. 130.

61 Robert S. Morison, “The biology of behavior,” *Natural History Magazine*, November 1975, p. 88–89. The quotation continued: “One of the arrows, incidentally, may be fired at rather close range by a young, energetic, and already celebrated population geneticist who has even more interest in social (and political) affairs than Wilson himself.”

62 Elizabeth Allen, Barbara Beckwith, Jon Beckwith, Steven Chorover, David Culver, Margaret Duncan, Steven Gould, Ruth Hubbard, Hiroshi Inouye, Anthony Leeds, Richard Lewontin, Chuck Madansky, Larry Miller, Reed Pyeritz, Miriam Rosenthal and Herb Schreier, “Against Sociobiology,” *New York Review of Books*, November 13, 1975, p. 43.

63 Elizabeth Allen, Barbara Beckwith, Jon Beckwith, Steven Chorover, David Culver, Margaret Duncan, Steven Gould, Ruth Hubbard, Hiroshi Inouye, Anthony Leeds, Richard Lewontin, Chuck Madansky, Larry Miller, Reed Pyeritz, Miriam Rosenthal and Herb Schreier, “Against Sociobiology,” *New York Review of Books*, November 13, 1975, p. 43–44.

64 John Tyler Bonner, “A new synthesis of the principles that underlie all animal societies,” *Scientific American*, October 1975, p. 129. Van den Berghe described it as an “infant discipline” (Van den Berghe 1976, p. 733) and West-Eberhard saw its “conceptual awkwardness” as a result of its “adolescent state” (West Eberhard 1976, p. 92).
